# Identification and mapping of new genes for resistance to downy mildew in lettuce

**DOI:** 10.1007/s00122-020-03711-z

**Published:** 2020-10-31

**Authors:** Lorena Parra, Kazuko Nortman, Anil Sah, Maria Jose Truco, Oswaldo Ochoa, Richard Michelmore

**Affiliations:** The Genome Center, Department of Plant Sciences, University of California, Davis, CA 95616 USA

## Abstract

**Key message:**

Eleven new major resistance genes for lettuce downy mildew were introgressed from wild *Lactuca* species and mapped to small regions in the lettuce genome.

**Abstract:**

Downy mildew, caused by the oomycete pathogen *Bremia lactucae* Regel, is the most important disease of lettuce (*Lactuca sativa* L.). The most effective method to control this disease is by using resistant cultivars expressing dominant resistance genes (*Dm* genes). In order to counter changes in pathogen virulence, multiple resistance genes have been introgressed from wild species by repeated backcrosses to cultivated lettuce, resulting in numerous near-isogenic lines (NILs) only differing for small chromosome regions that are associated with resistance. Low-pass, whole genome sequencing of 11 NILs was used to identify the chromosome segments introgressed from the wild donor species. This located the candidate chromosomal positions for resistance genes as well as additional segments. F_2_ segregating populations derived from these NILs were used to genetically map the resistance genes to one or two loci in the lettuce reference genome. Precise knowledge of the location of new *Dm* genes provides the foundation for marker-assisted selection to breed cultivars with multiple genes for resistance to downy mildew.

**Electronic supplementary material:**

The online version of this article (10.1007/s00122-020-03711-z) contains supplementary material, which is available to authorized users.

## Introduction

Downy mildew, caused by the oomycete *Bremia lactucae*, is the most important disease in lettuce (*Lactuca sativa* L.) worldwide. *B. lactucae* is a biotrophic pathogen that primarily infects the foliar tissue, reducing yield and decreasing the quality of the marketable portion of the crop. The use of cultivars carrying dominant resistant genes (*Dm* genes) is the most effective strategy to control this disease. However, *B. lactucae* rapidly evolves to new virulence phenotypes that defeat individual *Dm* genes. For this reason, lettuce breeding programs continually seek new sources of resistance to downy mildew and new resistance genes (Crute 1992; Lebeda et al. 2014; Parra et al. 2016).

For over fifty years, breeding for resistance to *B. lactucae* has relied on the introgression of new genes from wild species (Crute 1992; Beharav et al. 2006; Parra et al. 2016). Of the 100 wild *Lactuca* species described thus far, only 14 are known to be natural hosts of *B. lactucae* (Lebeda et al. 2002). *L. serriola* and *L. saligna* have been used as sources of resistance to downy mildew in multiple breeding programs. *L. virosa* also possesses race-specific resistance to *B. lactucae,* but its use in breeding programs has been restricted due to infertility barriers between *L. virosa* and *L. sativa*; however, interspecific crosses followed by embryo rescue have occasionally allowed the transfer of resistance from *L. virosa* to *L. sativa* (Maisonneuve et al. 1999; Maisonneuve 2003). Introgression of resistance from *L. serriola*, *L. saligna*, and *L. virosa* through repeated backcrosses to *L. sativa* has been carried out by several public and commercial breeding programs resulting in a large increase in the number of resistance genes being deployed (Michelmore et al. 2013).

The genome of *L. sativa* cv. Salinas has been sequenced and assembled (Reyes-Chin-Wo et al. 2017). The latest assembly covers 2.4 Gb of the total estimated 2.7 Gb lettuce genome; these sequences are ordered in nine chromosomal pseudomolecules (Reyes-Chin-Wo et al. 2017). The 27 known *Dm* genes are located in major resistance clusters (MRC) that contain genes encoding nucleotide-binding leucine-rich repeats (NLRs) (Christopoulou et al. 2015a, b). Currently, over 50 genes for resistance to lettuce downy mildew have been reported and genetically characterized to varying extents (reviewed in Parra et al. 2016). While 27 *Dm* genes have been mapped to individual clusters in the reference genome, another 23 resistance factors remain uncharacterized (Parra et al. 2016). The constant advances in genome sequencing technologies have increased the amount of sequence data generated, while the cost of sequencing continues to decrease; this enables the use of whole-genome sequencing (WGS) as a tool to compare different genotypes, find introgressed regions with high divergence, and indicate candidate positions of resistance genes.

In this study, we used WGS of 11 near isogenic lines (NILs) and recurrent genotypes to identify the chromosome segments introgressed from wild donor species. Segregating populations derived from these NILs were used to position the new resistances on the reference genome of lettuce. The knowledge of the position of these new genes for resistance to lettuce downy mildew will facilitate marker-assisted selection to pyramid multiple *Dm* genes into cultivars, which will increase the evolutionary hurdle for the pathogen to become virulent and provide more durable disease resistance.

## Methods

### Plant materials

Multiple advanced breeding lines have been generated during the last four decades as products of the lettuce breeding program at UC Davis. The 11 NILs used in this study had been previously generated from crosses of wild *Lactuca* accessions and *L. sativa* cv. Salinas, followed by six backcrosses to cv. Salinas (Table 1). F_2_ populations were derived from crosses of each NIL (male) with *L. sativa* cv. Salinas (female).

**Table 1:**
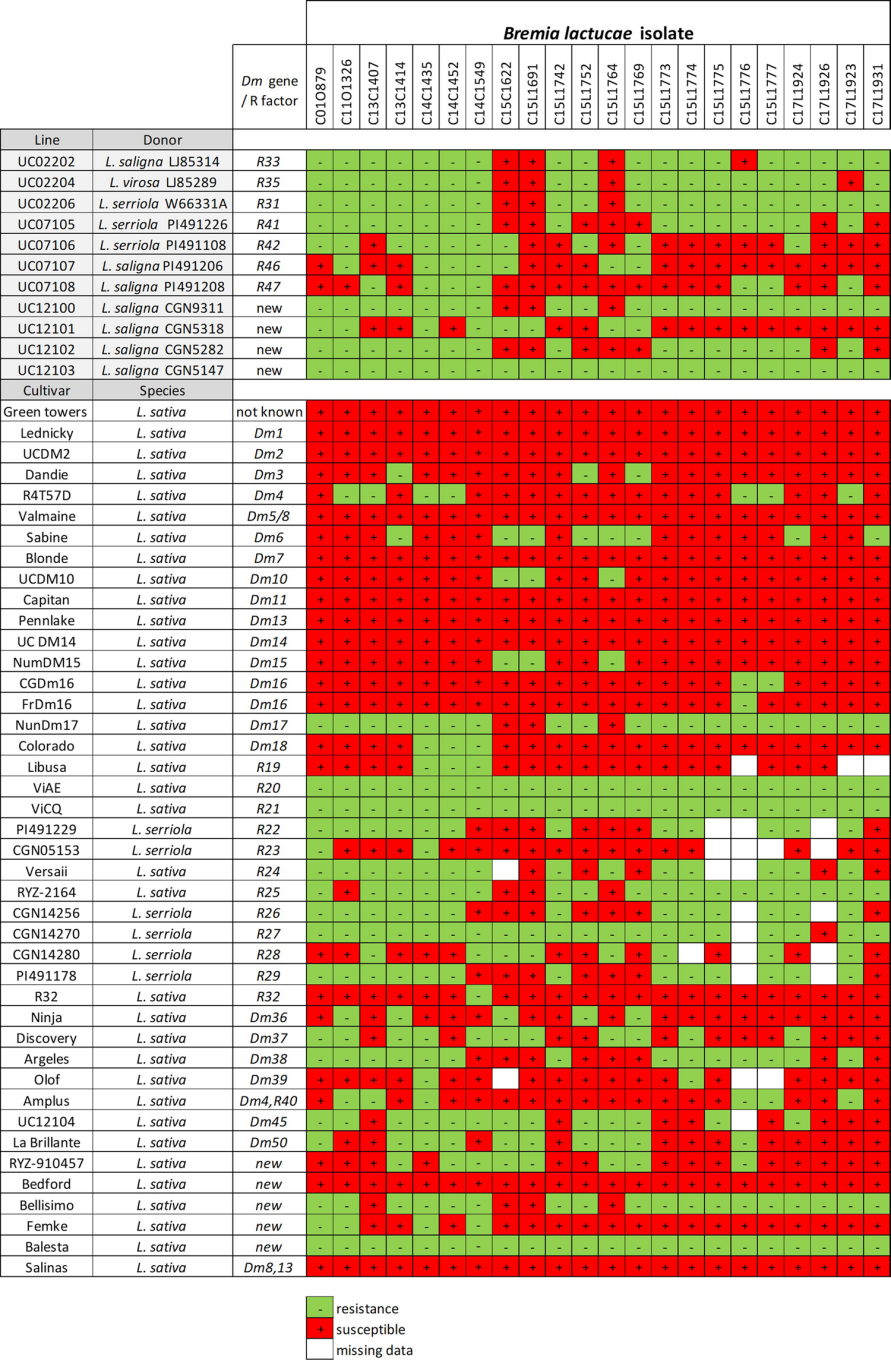
Resistance specificities to multiple isolates of *B. lactucae* in near isogenic lines (NILs) with introgressed chromosomal segments from wild species of *Lactuca*

Green/negative = resistance; red/plus = susceptible. *L. sativa* cv. Salinas (recurrent parent) is susceptible to all isolates used in this study

### Low-pass whole genome sequencing

DNA was extracted from seedlings of each NIL using GeneElute™ Plant DNA extraction miniprep kit (Sigma-Aldrich, MO, USA). DNA was fragmented using a COVARIS^R^ sonicator in order to obtain an average fragment size of 350 bp. Genomic DNA libraries for individual NILs were made using KAPA hyper prep kit for Illumina sequencing (KAPA Biosystems, MA, USA). Libraries were size selected for a range of 300–400 bp using BluePippin (Sage Science, MA, USA), then barcoded, multiplexed, and sequenced using an Illumina Hi-seq 2500 platform (Illumina Inc., CA, USA).

### Determination of introgressed region

Read mapping, variant detection, and visualization of SNP density was done using CLC Genomics Workbench 11.0 (https://www.qiagenbioinformatics.com/). Reads from each NIL were mapped into the Salinas reference genome v8 (Reyes-Chin-Wo et al. 2017), using a minimum cut-off of 90% similarity. Single nucleotide polymorphisms (SNPs) were only called for regions with a minimum coverage of three reads and when present in 90% of the total number of reads at each position. To avoid non-specific variants, SNPs generated by mapping cv. Salinas reads against the cv. Salinas reference genome were filtered out. Illumina reads from cv. Salinas 88 (cv. Salinas + *mo* gene introgressed from *L. serriola* PI 251245) were used to set the optimal parameters for mapping and variant detection against the cv. Salinas reference genome.

### Tests for resistance to *Bremia lactucae*

NILs were evaluated using seedling-based tests for resistance to multiple isolates of *B. lactucae* from California. All isolates were grown on seedlings of *L. sativa* cv. Green Towers, which expresses no known genes for resistance to downy mildew. Spores were collected in distilled water after 7 days and used immediately for inoculations. Seedlings from NILs and 42 lettuce cultivars (Table 1) were grown in a growth chamber at 17 °C for 7 days and sprayed with a ~ 10^4^ spores/mL suspension of *B. lactucae*. Lines/cultivars with sporulation on more than 50% of the seedlings were considered susceptible; lines/cultivars with no sporulation were considered resistant. For the phenotyping of F_2_ individuals, cotyledons from 7-day-old seedlings were detached and placed over wet filter paper in 48-well cell culture plates and then inoculated with ~ 25 µL of a suspension of *B. lactucae* spores. Cotyledons with full sporulation covering the whole leaf area were considered susceptible, while cotyledons with no sporulation were considered resistant. Cotyledons with sparse sporulation (only a few such cotyledons were observed) were not considered in the analysis. For the phenotyping of F_3_ families derived from F_2_ individuals, seedlings were inoculated following the same protocol used for the NILs. Resistance phenotypes for both seedlings and detached cotyledons were recorded 7 and 14 days post-inoculation.

### Genotyping and mapping of R-genes

A subset of the F_2_ populations derived from each NIL were genotyped using genotyping-by-sequencing (GBS) as described in Macias-González et al. (2019) (Table 2). DNA was extracted from ~ 50 seeds from each F_2_ plant, digested with *Ava*II, ligated to barcoded adapters, and multiplexed in pools ranging from 250 to 300 individuals, with cv. Salinas included in each set. Pools were size-selected to ~ 350 bp using BluePippin and paired-end sequenced using an Illumina Hi-seq 2500 platform (Illumina Inc., CA, USA). After demultiplexing, TASSEL3 was used to obtain individual genotypes (Glaubitz et al. 2014). Visualization and selection of SNPs was done using Microsoft Excel. The SNP genotypes were converted to alleles A, H, or B, where A was homozygous for the susceptible parent, B was homozygous for the resistant parent, and H was heterozygous. For the lines UC07106 and UC07108, standard interval mapping was performed using R-QTL (Broman et al. 2003). The significance threshold for the logarithm (base 10) of odds scores (LOD) for QTLs was calculated by 1000 permutations at the significance thresholds of α < 0.05 and α < 0.01.

**Table 2:** F_2_ segregation ratios and chromosomal positions of R-genes present in NILs

Line	Isolate (F2 phenotyping)	No. plants (R/S)	Seg. ratio	Chi-square	Genetics	Isolate (F3 phenotypimg)	No. of sequenced	R-gene position*	Dm gene
UC02202	C11O1326	105R/35S	3:1	0	1 Dominant	C15L1742	65	Chr1	Dm33
UC02204	C11O1326	107R/36S	3:1	0.002	1 Dominant	C15L1777, C17L1926	72	Chr1	Dm35
UC02206	C11O1326	109R/35S	3:1	0.037	1 Dominant	C17L1926	72	Chr1	R31
UC07105	C11O1326	105R/31S	3:1	0.352	1 Dominant	C15L1777	67	**MRC4**	R41
UC07106	C11O1326	90R/44S	~3:1	3.59	1 Dominant	C15L1752	96	**MRC1**	R42
UC07107	C11O1326	32R/97S	1:3	0.002	1 Recessive	C1101326	116	**MRC1**	Dm46
UC07108	C13O1407	68R/56S	9:7	0.1	2 Dominant	C15L1777	106	**MRC2**, **MRC4**	R47
UC12100	C11O1326	104R/32S	3:1	0.157	1 Dominant	C15L1742, C17L1926	72	Chr1	s.f.d (R60)
UC12101	C11O1326	81R/63S	9:7	0	2 Dominant	C15L1691	143	**MRC1**,** MRC2**	s.f.d (R64)
UC12102	C11O1326	109R/35S	3:1	0.037	1 Dominant	C15L1777	72	**MRC4**	s.f.d (R61)
UC12103	C11O1326	111R/33S	3:1	0.333	1 Dominant	C17L1926	72	Chr1	s.f.d (R62)
UC12103		22R/10S	3:1	0.666	1 Dominant	C15L1691	32	**MRC1**	s.f.d (R63)

Fragments co-located with MRC clusters are shown in bold letters (*based on the reference genome of cv. Salinas).* R/S* resistant/susceptible. Chi-square calculated according to Σ (observed value-expected value)^2^/expected value. s.f.d: submitted for denomination

### Availability of data and materials

Phenotypic and genotypic datasets for the progenies are available upon request.

## Results

### Screening of NILs for resistance to multiple *B. lactucae* isolates from California

Eleven NILs and another 42 lettuce cultivars carrying known *Dm* genes were evaluated using seedling-based tests for resistance to 25 isolates of *B. lactucae* from California. Seven of these NILs were resistant to most of the isolates tested, while the other four NILs were resistant to at least ten *B. lactucae* isolates (Table 1). Several NILs showed patterns of resistance consistent with the expression of new *Dm* resistance genes. Interestingly, the NILs UC02206 and UC12100 displayed the same resistance specificity as the line NunDm17, which carries *Dm*17 (Table 1), and the NILs UC07105 and UC12102 exhibited the same resistance profile. UC12103 was resistant to all tested isolates. Only one lettuce cultivar, Balesta, was resistant to all tested isolates (Table 1). Two lines with resistance from *L. virosa*, ViAE and ViCQ, were also resistant to all isolates tested.

### Determination of introgressed regions in NILs using low-pass whole genome shotgun sequencing data

**Fig. 1:**
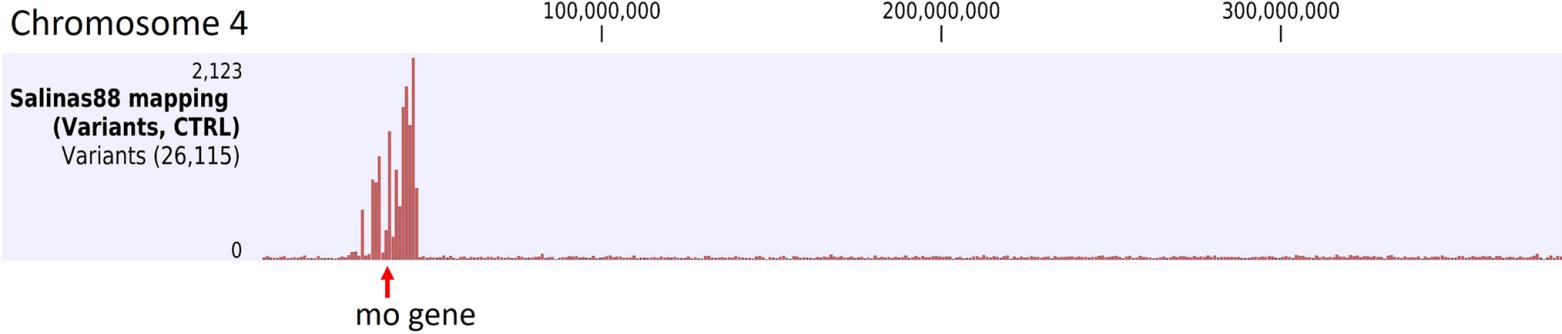
Detection of introgression in Salinas 88. Detection of SNP variants against the *Lactuca sativa* cv. Salinas reference genome shows a highly polymorphic small region in Chromosome 4 (red bars). The *mo* gene is located in the middle of the introgressed region

*L. sativa* cv. Salinas that was used as a recurrent parent for the generation of the NILs was also sequenced to assemble the reference genome (Reyes-Chin-Wo et al. 2017). This facilitated the identification of introgressed regions in the NILs by visualizing chromosomal regions with high densities of SNPs when comparing reads of the NILs aligned against the reference genome. This approach was validated using cv. Salinas 88, which contains the *mo* gene for resistance to lettuce mosaic virus introgressed from *L. serriola* PI251245 (Ryder 1991; Mikel 2007) into cv. Salinas. Salinas 88 reads were aligned against the Salinas reference genome and a single small (16 Mb) introgressed segment was identified on Chromosome (Chr) 4, which had previously been shown genetically to contain the *mo* gene (Fig. 1; Irwin et al. 1999). Salinas 88 was sequenced to 2.2×; analysis with different levels of coverage determined that the minimal average read coverage needed to accurately detect the size of the introgressed segment in cv. Salinas 88 was 0.5×.

**Fig. 2:**
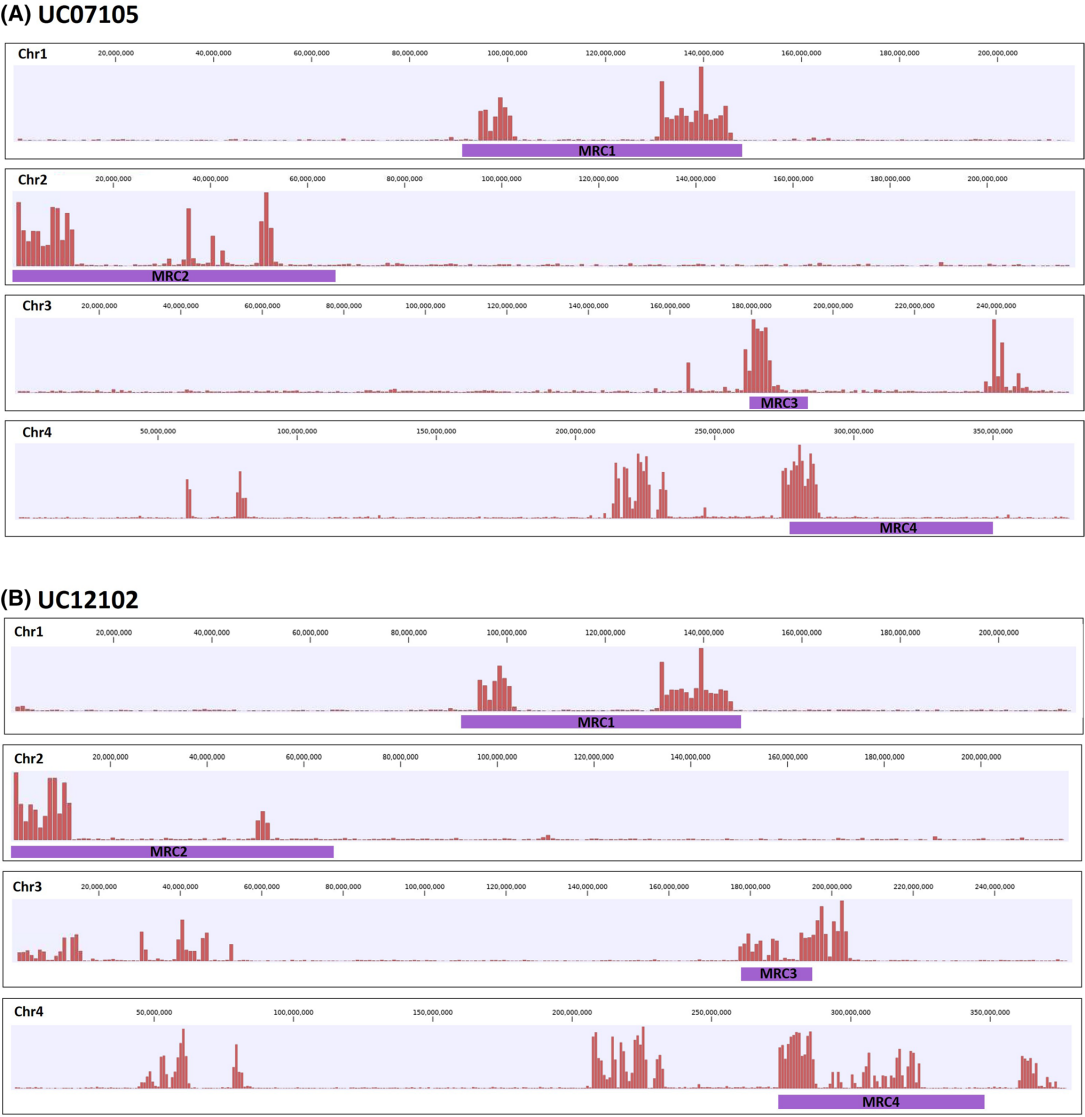
Example of introgressions in near isogenic lines (NILs) that are co-located with MRCs.** a** Introgressions for line UC07105;** b** introgressions for line UC12102. SNP density is indicated with red bars. Purple bars indicate the MRC regions in the reference genome

We were able to identify introgressed regions in all of the NILs. The NILs used in this study had been sequenced to an average read coverage of 1.1 × when mapped against the cv. Salinas reference genome. Some breeding lines had only a few separate segments with a high density of SNPs, while others contained multiple polymorphic regions (Supplementary Fig. 1). All of the NILs showed potential introgressions of regions encoding one or more MRCs (Supplementary Fig. 1). Most of the lines had introgressions of MRC1 and MRC2; only a few NILs had introgressions of MRC3 and MRC4 (Fig. 2). The SNP profiles are similar among some lines at MRC1 or MRC2; the reason for this is unclear but may reflect uneven recombination across the genome following interspecific crosses.

### Genetic mapping of new genes for resistance to downy mildew in lettuce

To identify which of the introgressed segments in the NILs are involved in DM resistance, F_2_ populations derived from ten NILs (UC02202, UC02204, UC02206, UC07105, UC07106, UC07107, UC12100, UC12101, UC12102, and UC12103) were screened for resistance to *B. lactucae* isolate C11O1326. The F_2_ population derived from UC07108 was screened for resistance to *B. lactucae* isolate C13C1407 rather than C11O1326 because the latter isolate was virulent on UC07108. The F_2_ populations derived from UC02202, UC02204, UC02206, UC07105, UC07106, UC12100, UC12102, and UC12103 showed a segregation ratio of 3:1 between resistant and susceptible individuals, consistent with one dominant resistant gene conferring resistance to downy mildew. The F_2_ populations derived from UC07108 and UC12101 showed a segregation ratio of 9:7, consistent with two dominant genes being required for resistance. The population derived from UC07107 had a segregation ratio of 1:3 between resistant and susceptible individuals, consistent with the presence of a recessive resistance gene.

**Fig. 3:**
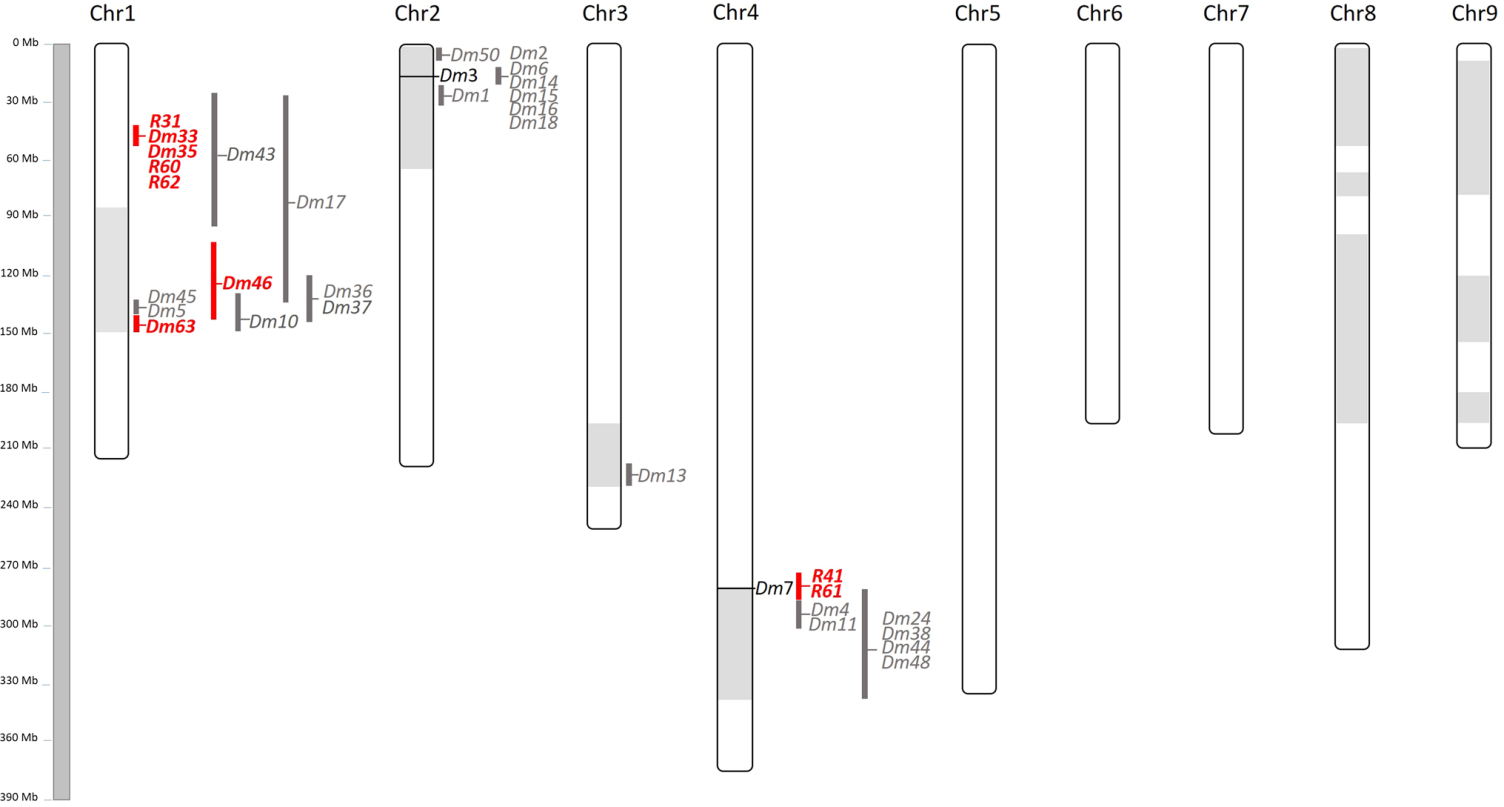
Graphic representation of lettuce Chromosomes 1, 2, 3, and 4 with the genomic location of new and known resistance genes. Genes in red letters were mapped in this study. Gray regions show the size intervals of the major resistance clusters MRC1, MRC2, MRC3, and MRC4

F_2_ populations were genotyped using GBS and resistance genes were positioned on the reference genome (Table 2, Fig. 3, and Supplementary Table 1). From the nine populations with simple Mendelian segregation (3:1), six had resistance that mapped to Chr 1, while two populations had resistance that mapped to Chr 4. The recessive resistance to *B. lactucae* isolate C11O1326 present in UC07107 was positioned inside MRC1, while the resistance to the same isolate present in UC07105 and UC12102 was positioned inside MRC4. For the resistance genes present in the F_2_ populations derived from UC07108 and UC12101, which had segregation ratios of 9:7, the resistances were mapped to MRC2 and MRC4, and MRC1 and MRC2, respectively. The resistances to C11O1326 present in UC02202, UC02204, UC02206, UC12100, and UC12103 mapped outside the MRC regions (Fig. 3). For line UC07106, a region in Chr 1 was significantly associated with resistance.

Phenotyping seedlings of F_3_ families allowed us to narrow down the mapping interval for resistance genes from NILs UC07105, UC07106, UC12100, and UC12102 (due to the ability to phenotypically distinguish homozygous and heterozygous F_2_ resistant lines) to under half of the size detected in the F_2_. Additionally, by using F_3_ families we were able to test the resistances found in the F_2_ populations against another four *B. lactucae* isolates (C15L1742, C15L1752, C15L1777, C17L1926, and C15L1691). We found that the resistance genes detected in nine NILs can also confer resistance to at least one additional *B. lactucae* isolate. Moreover, we found an additional resistance gene in UC12103 that confers resistance to C15L1691, which was positioned inside MRC1.

### Denomination of the new *Dm* genes

The resistance identified in UC02202, UC02204, UC07105, UC07106, UC07107, and UC07108 was derived from donors with R-factors previously described in Parra et al. (2016); therefore, the *Dm* genes in these lines were named according to the R-factor denominations previously assigned to these donors (Table 2). However, until the uniqueness of the resistance specificity in UC02206 and UC07105 has been proven, the R-genes in these lines will remain R-factors. The same criteria was applied to the resistance genes in UC12100, UC12102, and the two new *R* genes in UC12103, which have been submitted to the International Bremia Evaluation Board (IBEB) for denomination as *R*60, *R*61, *R*62, and *R*63, respectively (Table 2). Genes in UC07108 and UC12101 will be kept as resistance factors (R), until we know which of the two genes in each line is the actual R-gene.

## Discussion

The genetic mapping of resistance genes in lettuce at high resolution can be challenging. Previously, *Dm* genes have been mapped using segregating populations, mostly F_2_ populations (Hulbert and Michelmore 1985; Farrara et al. 1987; Maisonneuve et al. 1994; McHale et al. 2009; Jeuken et al. 2009; Simko et al. 2015), allowing the determination of the map position for 27 *Dm* genes (Fig. 3) (Parra et al. 2016). However, the mapping resolution for most of these genes comprises windows from 10 to 70 Mb (McHale et al. 2009, Christopoulou et al. 2015b). In this study, we mapped an additional 11 *Dm* genes to different positions in the *L. sativa* cv. Salinas reference genome, six of them to genomic intervals no larger than 6 Mb (Fig. 3, Supplementary Table 1). The highest resolution was the localization of the resistance gene in UC12102 to a 3.4 Mb window.

Several NILs used in this study had unique resistance profiles against the range of *B. lactucae* isolates tested. The line UC12103 showed resistance to all 25 isolates tested. The lines Balesta, VIAE, and VICQ were also resistant to all tested isolates. However, the genomic positions of the resistances present in these lines have not been reported, which makes it impossible to determine whether they share the same gene(s), or whether the observed phenotype is due to a combination of several *Dm* genes that recognize multiple *Avr* proteins from these isolates of *B. lactucae*. Interestingly, the NILs UC02206 and UC12100 displayed the same resistance profile as NunDm17, which carries *Dm*17, despite the fact their resistances were introgressed from different wild sources (*L. serriola* W66331A, *L. saligna* CGN9311, and *L. serriol*a LS102, respectively). Both resistances present in these lines mapped to Chromosome 1, overlapping the genomic region where *Dm*17 had been previously mapped (Maisonneuve et al. 1994; Mc Hale et al. 2009) (Fig. 3). Similarly, the lines UC07105 and UC12102 had identical resistance specificities to each other and their resistances mapped to the same region on Chromosome 4, despite both resistances being introgressed from different *Lactuca* species (*L. serriola* PI491226 and *L. saligna* CGN5282, respectively). Different lettuce genotypes with the same resistance specificity to isolates of *B. lactucae* have been observed before. An example of this is *Dm5* in *L. serriola* PI167150 and *Dm8* in *L. serriola* PI104584. *Dm5* and *Dm8* confer resistance to the same spectrum of isolates and map to the same genetic position (Norwood and Crute 1984; Hulbert and Michelmore 1985). Future cloning of the genes present in the NILs will clarify whether these lines carry the same *Dm* gene or different *Dm* genes with the same downy mildew resistance specificity.

### Genome introgressions from wild Lactuca spp.

Whole genome sequencing and variant detection is a successful and low-cost method to identify introgressions from wild species (Severin et al. 2010). However, the polymorphic regions visualized on the cv. Salinas reference genome may not necessarily represent the real size of the introgressed segments from the wild donors in the NILs. Alignment to a reference genome limits the possibility of displaying regions with extremely high divergence or regions that have been deleted/inserted in the chromosomal segment of the wild donor. Some introgressed segments were very small in the NILs, as expected in lines that have been backcrossed multiple times to the recurrent parent. However, some lines had extremely large chromosomal segments introgressed from the wild parent. Low recombination rates can be a product of highly divergent regions between cultivated and wild type *Lactuca* spp. (Liharska et al. 1996).

For UC07106, a ~ 40 Mb region in MRC1 was significantly associated with resistance to isolate C11O1326 (Supplementary Fig. 2), although not all the susceptible individuals had a homozygous susceptible genotype, indicative of incomplete penetrance of this resistance. Phenotyping of F_3_ families followed by QTL mapping narrowed down the region to 31 Mb and confirmed the discrepancy between the susceptible genotype and phenotype; F_3_ data also demonstrated cosegregation of resistance to an additional *B. lactucae* isolate, C15L1752 (Supplementary Fig. 2). Segregation in the Salinas x UC07106 F2 population deviated from simple Mendelian expected ratios; however, the closest fit was to a 3:1 segregation ratio, consistent with the presence of a dominant resistance gene. According to the SNP density profiles, the UC07106 line shows an introgression in Chromosome 1 that replaced more than half of the chromosome, including the MRC1 (Supplementary Fig. 1). This could indicate high divergence in this region between cv. Salinas and *L. serriola* PI491108 (recurrent and resistant donor for this NIL, respectively). Therefore, we hypothesize that the resistance gene in line UC07106 is located in a chromosomal region that is not present in the reference genome. Rearrangement of Chromosome 1 has been observed in *L. sativa* cv. Tizian, which showed a large region that is either missing or highly divergent from MRC1 in the cv. Salinas reference genome (Verwaaijen et al. 2018).

### Different genetic basis for resistance to *B. lactucae*

Two NILs, UC07108 and UC12101, had an F_2_ segregation ratio that indicates that two dominant genes are required for the resistance phenotype. This could be indicative of the presence of one NLR R-gene that requires an additional gene for either recognition of pathogen effector or signaling of the defense response (Warren et al. 1999). One possibility is that two NLR genes are acting as a pair, with one of the NLR genes acting as a sensor NLR and the other as executor NLR (Jubic et al. 2019). However, a characteristic feature of NLR pairs in other species is that both members are often neighbors in inverse orientation in the genome and therefore genetically co-segregate (Oyuyama et al., 2011; Cesari et al., 2013; Baggs et al., 2017; Ortiz et al., 2017). The two genes in both lines are genetically unlinked, making it unlikely for them to be NLR pairs. For line UC12101, one gene was mapped to Chr1 with multiple markers to support this location. However, the second gene was mapped to Chr2, with only a few markers to support this location. Meticulous observation of mapping reads at the MRC2 disproved the lack of polymorphism as a reason for the low number of markers found in this region. This could indicate that the wild lettuce donor and the recurrent parent had extremely divergent regions at the beginning of the MRC2, which complicated the selection of SNP markers in this region.

Only one of the NILs used in this study (UC07107) showed an F_2_ segregation ratio of 1:3, resistance to susceptibility, indicating the presence of a recessive gene. This ratio was observed regardless of the inoculum concentration or temperature; therefore, this phenotype was not due to incomplete penetrance. The selection of resistance individuals at each backcross generation during the generation of the NILs makes the selection of a recessive gene improbable; this line may have a linked dominant gene for resistance to other isolates that were used to test backcross progeny, resulting in linkage drag of the recessive resistance gene. The isolate used for phenotyping will directly influence the R-gene identification. A clear example of this was line UC12103, where we were able to map two dominant resistance genes to different regions of Chr1, depending on the isolate used for the phenotyping of the F_2_ Salinas x UC12103 population (Table 2, Supplementary Fig. 3).

### Implications for breeding and future directions

**Fig. 4:**
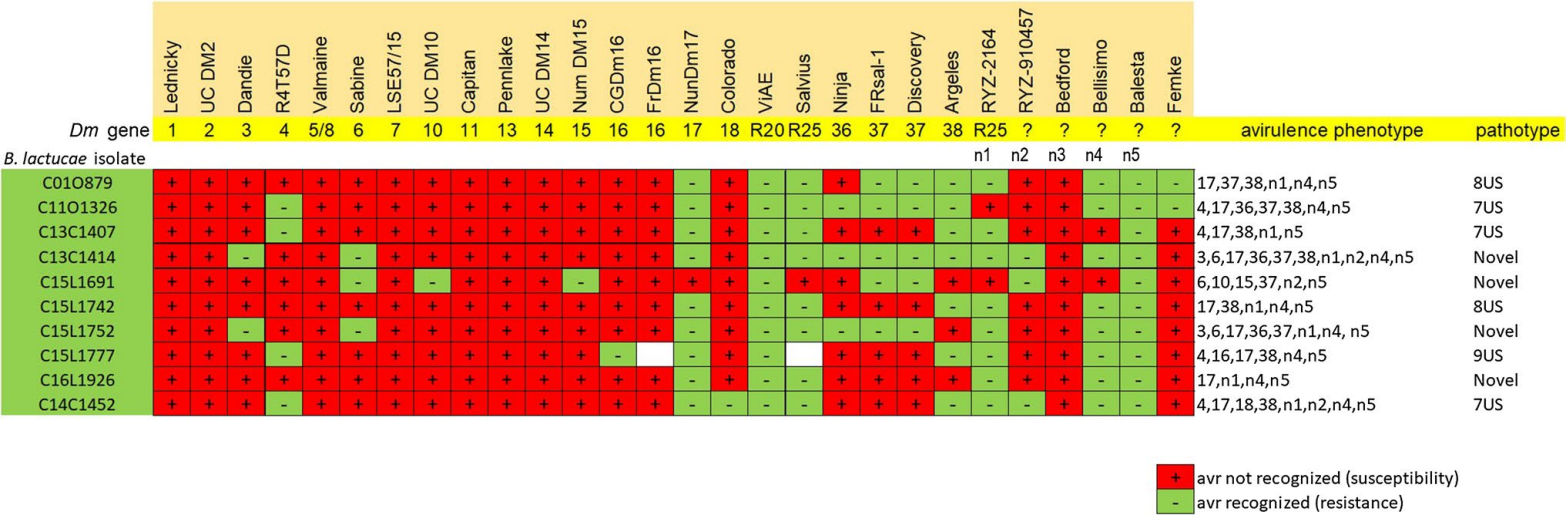
Virulence phenotypes and races of isolates of *B. lactucae* detected by the resistance genes identified in this study

The resistance genes detected in the lines UC02202, UC02204, UC02206, UC07105, UC07106, UC07107, UC07108, UC12100, UC12101, UC12102, and UC12103 confer resistance to races Bl:7US, Bl:8US, Bl:9US, and three isolates with novel virulence phenotypes (Fig. 4), representing some of the most prevalent virulence phenotypes in California over the past 10 years. These lines have been made available for lettuce breeding programs. However, multiple *Dm* genes should be combined to maximize the evolutionary hurdle for the pathogen to become virulent, to generate more durable resistance against *B. lactucae*. This will require multiple intercrosses and the use of molecular markers that identify the genomic regions identified in this study.

Most of the resistance genes identified in this study were mapped to small genomic intervals in different NILs. Sequencing the repertoire of NLR-encoding genes from these lettuce lines will allow us to find candidate genes for resistance to multiple isolates of *B. lactucae*. Cloning of the *Dm* genes present in these lines will provide the foundation for the development of stacks of *Dm* genes at single genomic positions using genome editing technologies. This will overcome the challenge of combining resistance genes derived from different sources that map to the same region. Stacking multiple *Dm* genes will greatly simplify breeding for more durable disease resistance because multiple *Dm* genes will be inherited as a single Mendelian locus.

## Electronic supplementary material

Below is the link to the electronic supplementary material.

Supplementary file 1 (DOCX 1441 kb)
