# Supplementary material for: Identification and mapping of new genes for resistance to downy mildew in lettuce
Source: Theor Appl Genet. 2020 Oct 31;134(2):519–28. doi: 10.1007/s00122-020-03711-z (PMC7843477; doi:10.1007/s00122-020-03711-z)
Supplement: Supplementary file 1 — (DOCX 1441 kb) [file 122_2020_3711_MOESM1_ESM.docx]

**Supplementary Figure 1. Multiple introgressions in different NILs, indicated by SNP density (red bars)**

**A. Chr1**
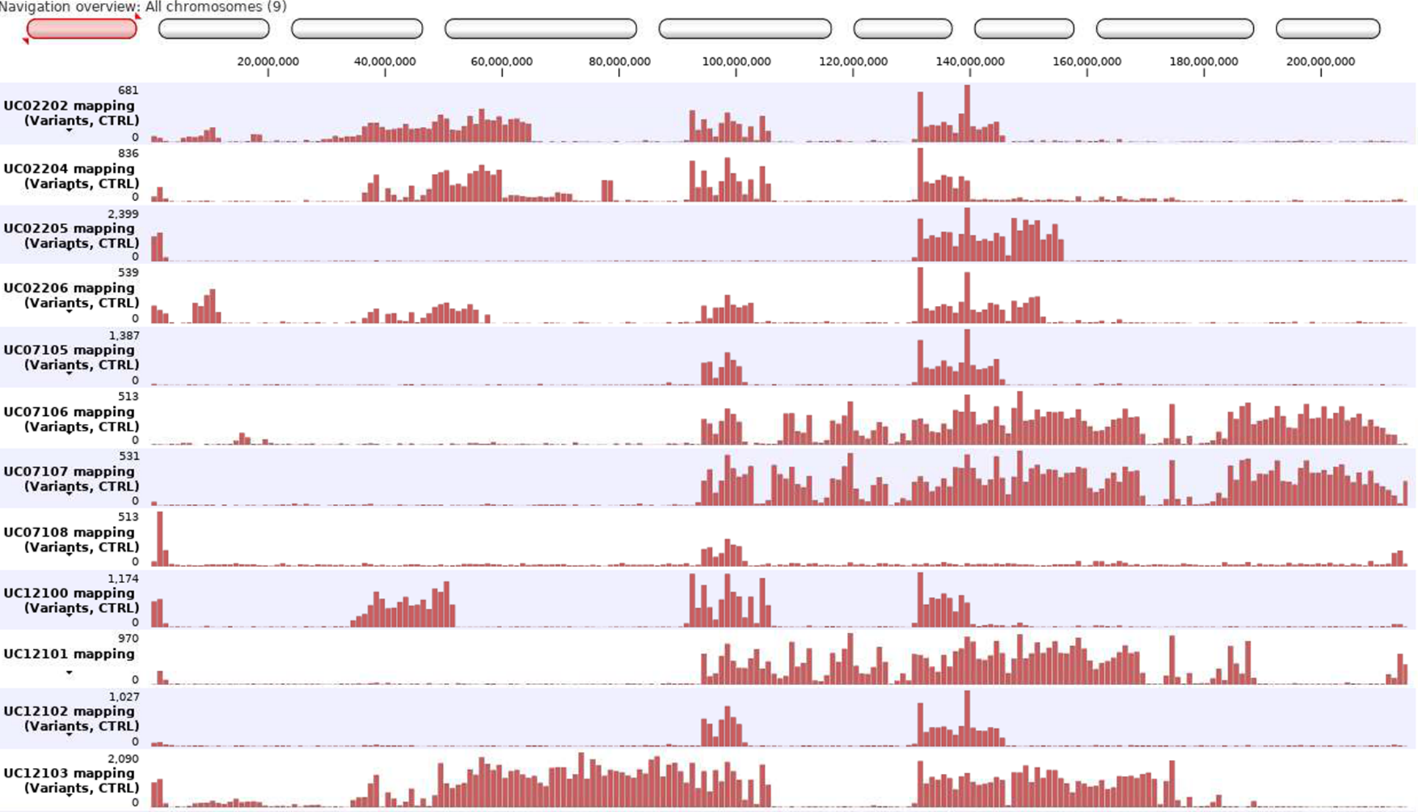


**B. Chr4
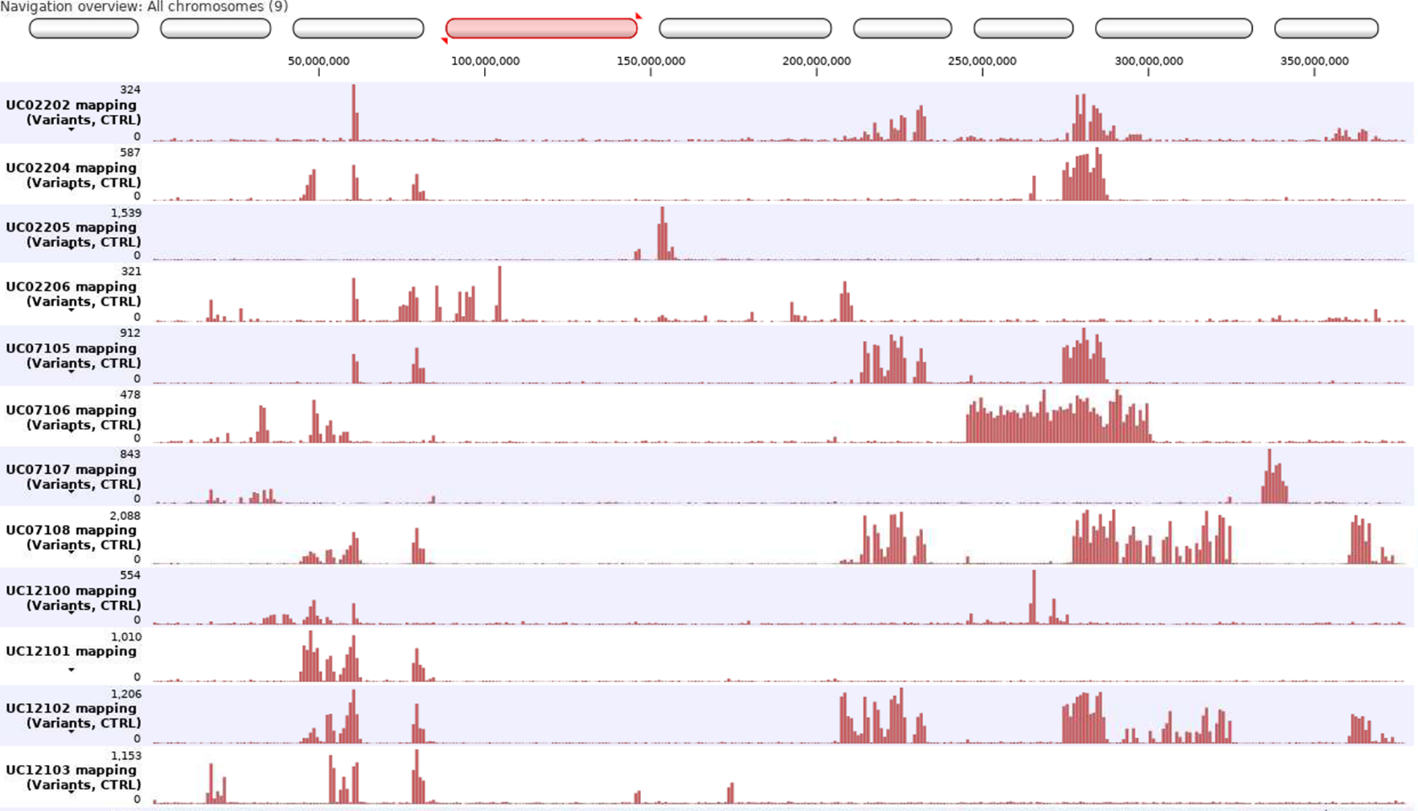
**

**Supplementary Figure 2. Mainscan LOD plot for Salinas x UC07106 F_2_ (A) and Salinas x UC07106 F_3_ (B).** The significance thresholds for the LOD scores at α ≤ 0.05 is represented with horizontal blue dotted lines.


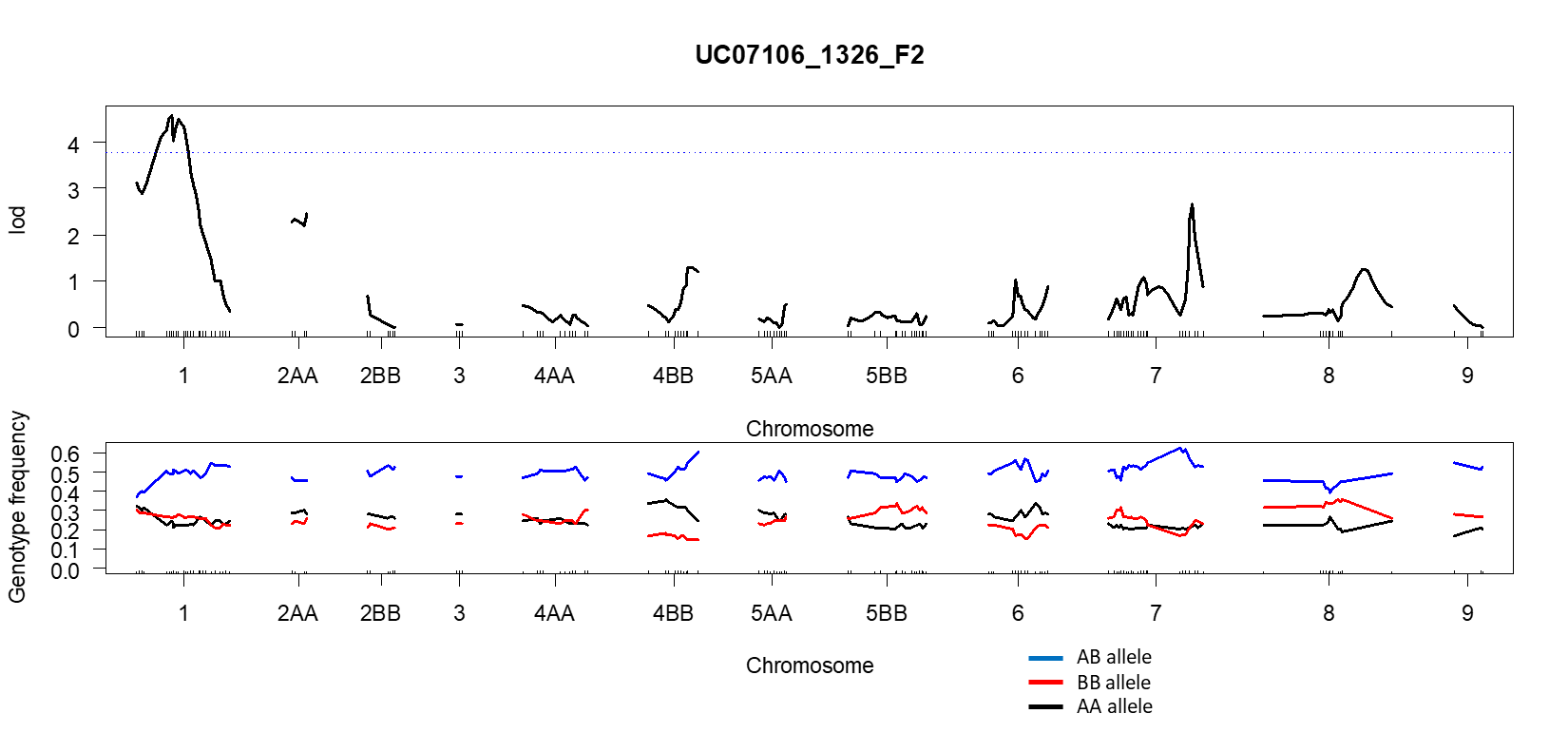


LOD interval (+/-1): 122-176 Mb in genome assembly v8, peak at 147 Mb.

Phenotype (evaluation of detached cotyledons from F_2_ plants) and F_2_ genotype at peak: Resistant: 50 (20 BB, 25 AB, 5 AA), Susceptible: 39 (3 BB, 19 AB, 17 AA)

**(B)**

**
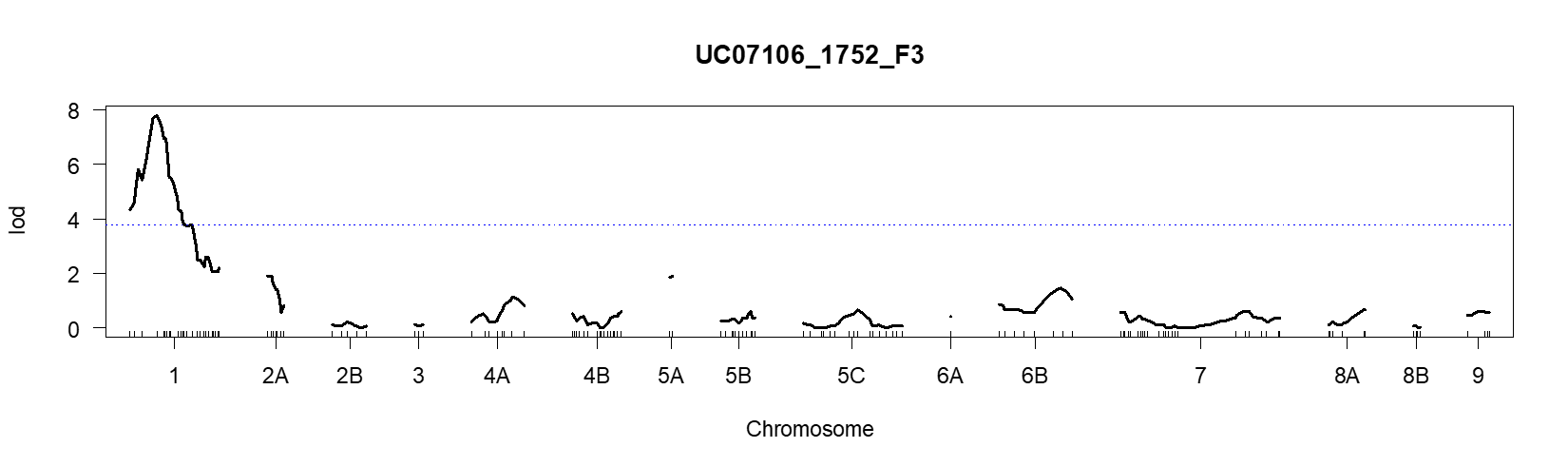
**

LOD interval (+/-1): 126-161 Mb in genome assembly v8, peak at 144 Mb

Phenotype (evaluation of 49 F3 families from resistant F2 plants) and F2 genotype at peak of QTL: Non-segregating F3 families: 28 (20 BB, 7 AB, 1 AA), Segregating F3 families: 21 (0 BB, 17AB, 4 AA)

**Supplementary Figure 3. Resistance genes in NIL UC12103**. Chromosome 1. Mapped intervals are highlighted in red. Green arrows indicate crossing over positions in Salinas x UC12103 F2 individuals. Blue boxes indicate NLR genes in the reference genome cv. Salinas.


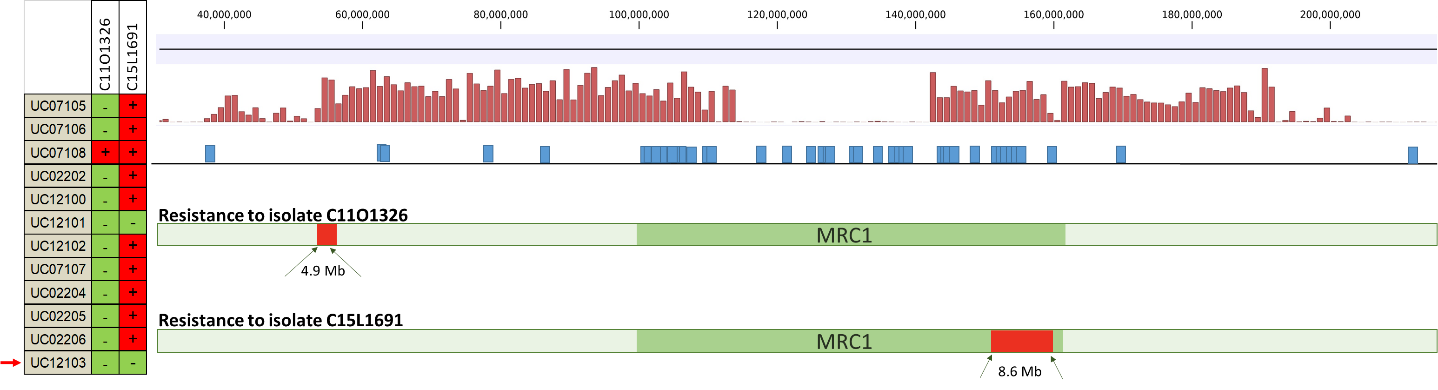


**Supplentary Table 1. Mapping interval for resistance genes present in NILS.** Resistance to isolates in bold was phenotyped in F_3_ families. Interval size in reference genome of cv. Salinas version 8.
